# TIM, a novel molecular toolbox for the detection and identification of *Leishmania* species in Morocco

**DOI:** 10.1128/spectrum.03447-25

**Published:** 2025-12-04

**Authors:** Imane El Idrissi Saik, Kaltoum Lemkhayar, Idris Mhaidi, Sara El Mazini, Soumiya Chiheb, Anne-Laure Bañuls, Meryem Lemrani, Myriam Riyad, Baptiste Vergnes

**Affiliations:** 1 Laboratory of Cellular and Molecular Pathology (Immunopathology of Infectious and Systemic Diseases team), Faculty of Medicine and Pharmacy, Hassan II University of Casablanca 92957 , Casablanca, Morocco; 2 Laboratory of Parasitology and Vector-Borne-Diseases, Institut Pasteur du Maroc 290327, Casablanca, Morocco; 3 MIVEGEC, Montpellier University, CNRS, IRD 27038, Montpellier, France; 4 Department of Dermatology, Ibn Rochd University Hospital 107900 , Casablanca, Morocco; Brown University, Providence, Rhode Island, USA

**Keywords:** *Leishmania*, diagnosis, genotyping, PCR, Morocco

## Abstract

Cutaneous leishmaniasis (CL) is the most prevalent form of leishmaniasis, with up to 1 million new cases reported annually across more than 90 endemic countries. In Morocco, CL is caused by three *Leishmania* species: *Leishmania major*, *L. tropica*, and *L. infantum*. Sensitive and specific parasite detection and accurate species identification are essential for proper diagnosis, treatment decisions, epidemiological studies, and effective disease surveillance. Commonly used PCR-based diagnostic methods require multi-step procedures, such as sequential amplification (Noyes nested PCR) or enzymatic digestion (ITS1-RFLP), and are not sufficiently resolutive for precise species identification. In this study, we introduce TIM (for *Tropica*, *Infantum*, *Major*), a novel, flexible, and straightforward molecular diagnostic tool based on standard PCR procedures. TIM targets specific motifs within kinetoplast DNA minicircle sequences, allowing for *Leishmania* detection in a single multiplex reaction. It can be adapted with a preliminary nested amplification step to enhance sensitivity or with species-specific primers for accurate species-level identification. When applied to CL clinical samples from Morocco, TIM demonstrated higher sensitivity than established methods. Notably, TIM also enabled the identification of mixed interspecific profiles directly from clinical samples, which may indicate either co-infections with distinct species or the presence of hybrid genotypes. With a streamlined protocol requiring only six primers, TIM is rapid, flexible, and well suited for both routine diagnostics and large-scale epidemiological investigations.

**IMPORTANCE:**

This study introduces TIM, a novel molecular diagnostic assay developed to enhance the detection and species identification of *Leishmania* parasites from clinical samples in Morocco. TIM relies on simple PCR procedures, and it is tailored to the detection of three species that are endemic to the region: *L. major*, *L. tropica*, and *L. infantum*. TIM demonstrated superior sensitivity and improved species-level resolution when compared to the diagnostic methods commonly used in Morocco. Notably, TIM also enables the direct detection of mixed interspecific profiles from cutaneous clinical samples. Thanks to its versatility and simplicity, TIM offers a practical tool to significantly strengthen leishmaniasis surveillance, prevention, and control strategies in Morocco and surrounding regions.

## INTRODUCTION

Leishmaniasis is a disease caused by *Leishmania* protozoa and transmitted to humans and animals through the bites of infected sandflies. These neglected tropical diseases present a spectrum of clinical manifestations ranging from self-healing cutaneous lesions (cutaneous leishmaniasis, CL) to severe and fatal visceral forms if untreated (visceral leishmaniasis, VL) (1). The Eastern Mediterranean region accounts for 83% of the CL cases reported worldwide (2). In Morocco, leishmaniases remain endemic, and most of the reported cases are cutaneous. In 2023, 2,359 CL and 79 VL cases were notified by the Moroccan Ministry of Health and Social Welfare (3). Three *Leishmania* species are responsible for infections in the country (*L. major*, *L. tropica*, and *L. infantum*), each with distinct epidemiological and clinical characteristics (4). CL caused by *L. major* is present in arid and semi-arid areas of the eastern and southern provinces, where it is characterized by alternating endemic and epidemic cycles (5). On the other hand, *L. tropica*, which is still considered anthroponotic, has the widest geographical distribution (6, 7). *L. infantum* is responsible for both CL and VL cases, primarily reported in the northern regions (8). Stray and domestic dogs are the main reservoirs of *L. infantum*. While domestic dogs maintain close contact with humans and are prone to re-infection, stray dogs act as mobile reservoirs that are difficult to monitor or treat, making control strategies more challenging. The epidemiology of leishmaniases in Morocco is therefore complex, significantly influenced by human mobility and climate change (9), which will further complicate efforts to control and manage the disease. In this context, early and accurate diagnosis of CL is crucial to prevent lesion aggravation, shorten treatment duration, enable timely outbreak response, or guide species-specific control and treatment strategies.

According to the WHO Road Map for Neglected Tropical Diseases (2021–2030), the development and deployment of improved diagnostic tools is a critical priority for achieving control and eventual elimination of CL (10). Current diagnostic needs are further outlined in the WHO Target Product Profile (TPP) for CL diagnostics, which emphasizes the importance of tests that are accurate and capable of species discrimination to guide treatment and surveillance (11). However, the diversity of *Leishmania* species causing CL, which mimic clinical presentations of other skin diseases, poses significant challenges. Traditional diagnostic methods, such as microscopic examination or parasite culture, have long been used but present several limitations, notably low sensitivity and the inability to differentiate *Leishmania* species (12). Molecular techniques, particularly PCR-based assays, have revolutionized leishmaniasis diagnosis by offering high sensitivity and specificity. PCR enables species identification, which can be critical for guiding treatment decisions, as different *Leishmania* species exhibit varying drug susceptibilities and clinical outcomes (13).

Among the various genetic markers used for PCR diagnostics, kinetoplast DNA (kDNA) and ribosomal DNA (rDNA), specifically the internal transcribed spacer (ITS) regions, are the most commonly targeted sequences due to their high copy number. The *Leishmania* rDNA consists of conserved coding regions (small subunit 18S rRNA and large subunit 28S rRNA) interspersed with more variable ITSs (ITS1 and ITS2). ITS1 (located between 18S and 5.8S rRNA) and ITS2 (between 5.8S and 28S rRNA) exhibit interspecies variation, making them good markers for *Leishmania* species identification. The ITS1-RFLP diagnostic approach consists of amplifying the ITS1 region by PCR and digesting PCR products with *Haemophilus aegyptius* endonuclease III (*Hae*III), which allows the identification of the different *Leishmania* species based on the restriction fragment sizes (14, 15). In addition, real-time quantitative PCR assays with melting curve analysis have been developed on the ITS1 locus, allowing the simultaneous detection and differentiation of groups of *Leishmania* species (16).

The kDNA consists of a network of thousands of concatenated circular DNA molecules present in the single mitochondrion of *Leishmania* parasites. It contains two types of circles: maxicircles (20–40 kb) and minicircles (~1 kb). Minicircles are present in high copy numbers (up to 10,000 per parasite), making them an excellent target for highly sensitive PCR assays. However, due to the high sequence heterogeneity within the variable domain of minicircles, primer design remains challenging, requiring a targeted approach toward the conserved domain for consistent amplification. This conserved domain of about 120 bp contains three conserved motifs of less than 10 bp (conserved sequence blocks CSB1-3), around which a number of tests have been developed (17). The Noyes nested-PCR test, developed in 1998, is one of the most widely used (18). Thanks to a nested step, it offers better sensitivity and works on both Old World and New World *Leishmania* species.

In practice, however, both ITS1-RFLP and Noyes nested PCR present several limitations, including time-consuming protocols and the need for a two-step open procedure, which increases the risk of cross-sample contamination (Table 1). We present TIM, a new flexible PCR-based toolbox inspired by the Noyes method on kDNA but optimized for greater accuracy in identifying *Leishmania* species circulating in Morocco. Thanks to its versatility, TIM can fulfill three distinct functions: a highly sensitive test with a nested amplification step (TIM1), a rapid batch screening based on a single PCR reaction (TIM2), and a high-resolution species discrimination and genotyping test (TIM3; Table 1). We validated our approach using clinical samples from autochthonous CL patients, demonstrating the reliability of TIM for parasite detection accuracy across various clinical sample types. While some scalability barriers remain, such as equipment requirements and the need for trained personnel, TIM provides a new sensitive and accurate alternative to the molecular assays commonly used in Morocco for both routine diagnosis and epidemiological surveillance, within a “One Health” framework.

**TABLE 1:** Comparison of conventional and TIM PCR-based assays for *Leishmania* detection and species discrimination^*b*^

PCR-based assays	First step	Second step	Species discrimination on agarose gels	Observations	Time to result^*a*^	Primer IDs and sequences (5′−3′)	DNA target	Ref
ITS1-RFLP	PCR amplification with primers LITSR + L5.8S	*Hae*III restriction of PCR products	Restriction profiles	Two steps open procedure discrimination between *Linf* and *Ltrop* based on a single additional band at 100 bp	3 h	LITSR: CTGGATCATTTTCCGATG L5.8S: TGATACCACTTATCGCACTT	rDNA	14
« Noyes » Nested PCR	First PCR amplification with internal primers CSB1XR and CSB2XF	Second PCR amplification with external primers LIR and 13Z	Sizes of amplified products	Two steps open procedure sizes discrimination can be difficult between *Linf* and *Ltrop* species	3 h 30 min	CSB1XR: ATTTTTCSGWTTYGCAGAACG CSB2XF: SRTRCAGAAAYCCCGTTCA 13Z: ACTGGGGGTTGGTGTAAAATAG LIR: TCGCAGAACGCCCCT	kDNA	18
TIM1 (optional)	First multiplex PCR amplification using primers T1R, T1F1, and T1F2	Second PCR amplification using either TIM2 or TIM3 assays	See TIM2 or TIM3 results	Two steps open procedure see TIM2 or TIM3 observations	3 h 30 min	T1F1: GTGCAGAAAYCCCGTTCA T1F2: SATACAGAAACCCCGTTCA T1R: GCAGAACGCCCCTACC	kDNA	This study
TIM2	Multiplex PCR with T2R1 + T2R2 + T2F primers		Sizes of amplified products	One step closed procedure sizes discrimination can be difficult between *Linf* and *Ltrop* species	2 h	T2F: CTRGGGGTTGGTGTAAAATAG T2R1: CGNRGGACCAGAAAAGTTTG T2R2: TACCCGGAGGGCTACTM	kDNA	This study
TIM3	PCR amplification with TIM2F + TIM2 R1 primers (for *Linf* and *Lmaj* species)		Sizes of amplified products	Two parallel one-step closed procedures identification of mixed genotypes	2 h	T2F: CTRGGGGTTGGTGTAAAATAG T2R1: CGNRGGACCAGAAAAGTTTG	kDNA	This study
	PCR amplification with TIM2F + TIM2 R2 primers (for *Ltrop*)		Presence/absence			T2F: CTRGGGGTTGGTGTAAAATAG T2R2: TACCCGGAGGGCTACTM		

^*a*^ Estimated time from DNA sample considering 1 h 30 min by PCR, 1 h for restriction enzyme digestion, and 30 min for gel electrophoresis.

^*b*^ The table summarizes the main features of the ITS1-RFLP, Noyes nested PCR, and TIM assays (TIM1–3), including workflow design, target DNA, primer sequences, and estimated time to result. *Linf*: *L. infantum*;* Ltrop*: *L. tropica*;* Lmaj: L. major*.

## MATERIALS AND METHODS

### Patient sampling

The samples used in this study were obtained from CL patients who sought healthcare at the Dermatology Department of the Ibn Rochd University Hospital Center in Casablanca, or were sampled in endemic localities between 2018 and 2024. The following sampling techniques were used in the study: skin dermal aspirates of exudates that were obtained from the suspected lesion border, skin biopsies, or swabs. Dermal samples were cryopreserved at −20°C until DNA extraction. The species identification was carried out by ITS1-PCR followed by *Hae*III RFLP. All the patients were treated for free either at Ibn Rochd University Hospital or at the respective health centers according to the recommendations of the Moroccan Ministry of Health.

### DNA extraction

Total DNA was extracted from biopsies, dermal aspirates, and skin scrapings of the suspected CL patients with the phenol-chloroform method (15) and conserved at −20°C until use. Reference strains of *L. major* (MHOM/MA/2018/LC22), *L. tropica* (MHOM/MA/2018/LC12), and *L. infantum* (MHOM/MA/2018/LC11) were included: frozen strains at −80°C were thawed and then cultured in RPMI 1640 medium for reference DNA strains extraction. The concentration of each DNA sample was quantified using Qubit 3.0 fluorometer (Life Technologies).

### ITS1-RFLP

As previously described in Schönian et al. (14), DNA samples were amplified by a PCR targeting the *Leishmania*-specific ribosomal unit ITS1 using the primer pair L5.8S and LITSR and the MasterMix PCR HOT START x-VITA (2×; Labbox). The following thermocycling conditions were used: initial denaturation at 94°C for 2 min, followed by 32 cycles of denaturation at 94°C for 20 s, annealing at 53°C for 30 s, and extension at 72°C for 1 min, and a final extension at 72°C for 6 min in a thermocycler (Eppendorf Mastercycler EP Gradient S). Next, 15 µL of PCR products was digested with the *Hae*III (New England Biolabs) at 37°C for 1 h. The species were identified according to the restriction fragment sizes observed*: L. major* (two bands at 220 and 127 bp), *L. tropica* (two bands at 220 and 50 bp), and *L. infantum* (three bands at 200, 100, and 50 bp).

### kDNA nested PCR

kDNA Nested PCR was performed following the original protocol described in Noyes et al*.* (18). The first PCR was conducted using the primers CSB2XF and CSB1XR, as well as the MasterMix PCR HOT START x-VITA (2×; Labbox), with the following thermocycling conditions: initial denaturation at 94°C for 2 min, followed by 32 cycles of at 94°C for 20 s, annealing at 55°C for 30 s, and extension at 72°C for 1 min, and a final extension at 72°C for 6 min in a thermocycler (Eppendorf Mastercycler EP Gradient S). For the second PCR, we used 1 µL of a 9:1 dilution of the first PCR product in the same conditions using the primers 13Z and LiR. Amplifying the variable region of *Leishmania* kDNA allows the identification of the three species on a 1% gel: *L. major* (560 bp), *L. infantum* (680 bp), and *L. tropica* (750 bp).

### TIM procedure

**Fig 1:**
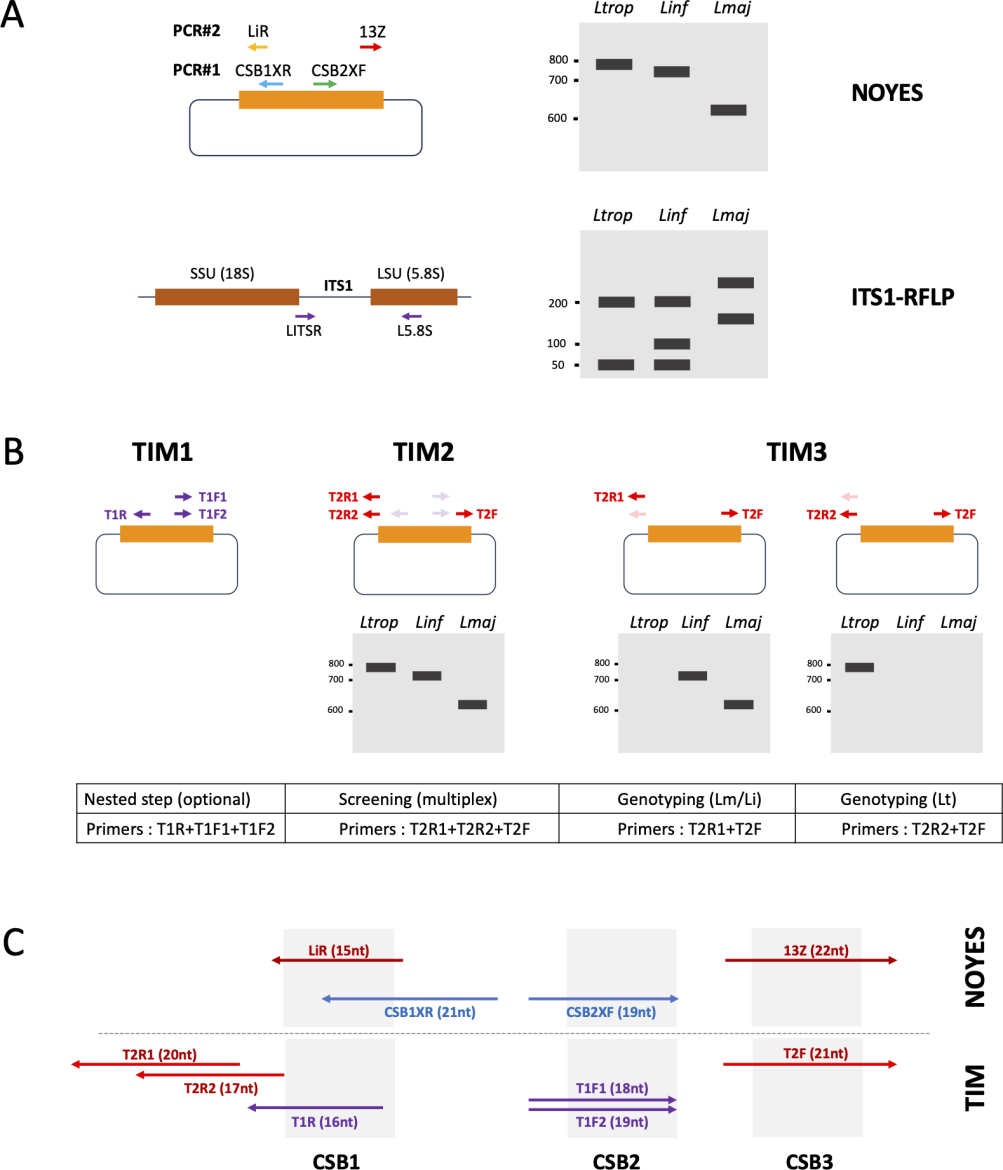
(**A**) Schematic representation of nested PCR on kDNA minicircles developed by Noyes et al. (18) and ITS1-RFLP on rDNA developed by Schönian et al. (14). Arrows represent primers’ location and names. Sizes of the expected amplification products (Noyes) or *Hae*III restriction fragments (ITS1-RFLP) are shown on the right. (**B**) Overview of the three TIM1, TIM2, and TIM3 tests with a schematic representation of a minicircle and its conserved domain (rectangle). Primers location and names are indicated with arrows. Virtual migration of amplified products is shown at the bottom. Ltrop, Lt: *L. tropica*; Linf, Li*: L. infantum*; Lmaj, Lm: *L. major*. (**C**) Sizes, names, and relative positions of primers used on both Noyes and TIM nested tests from the conserved domain of minicircles. CSB: conserved sequence block 1–3.

The global TIM procedure is illustrated in Fig. 1B. The amplification protocols for the three types of TIM PCRs—Nested PCR (TIM1), Multiplex PCR (TIM2), and Demultiplex PCR (TIM3)—are detailed below, using primers listed in Table 1. All PCR reactions were set up as follows: 10 µL of the Q5 Hot Start High-Fidelity 2× Master Mix (New England Biolabs), 8 µL of H_2_0, 1 µL of the corresponding Primer mix (see below), and 1 µL of the DNA sample. All PCR reactions were performed using the same cycling conditions with an optimized annealing temperature of 61°C for TIM2 and TIM3 and 62°C for TIM1: initial denaturation at 98°C for 2 min, followed by 32 cycles of 98°C for 10 s, 61°C (or 62°C for TIM1) for 20 s, and 72°C for 30 s, and a final extension at 72°C for 2 min. The primer mix for TIM1 nested PCR consists of primers T1F1, T1F2, and T1R, each at a final concentration of 10 µM. The primer mix for TIM2 multiplex screening PCR included primers T2R1, T2R2, and T2F at the same concentration (10 µM each final). For species discrimination, the TIM3 assay involved two separate reactions: one using primers T2R2 + T2F for *L. tropica* detection, and another using T2R1 + T2F to differentiate *L. major* and *L. infantum*. All PCR products were analyzed by electrophoresis on 0.7%–1% agarose gels stained with GelRed.

## RESULTS

### TIM development and procedure

Currently, ITS1-RFLP and Noyes PCR are the primary methods used in Morocco to confirm clinical suspicions of CL (both methods are summarized in Fig. 1A). The TIM test we propose is an adaptation of the Noyes assay, offering greater flexibility and specifically designed to improve detection efficiency for the three *Leishmania* species circulating in the country: *L. major*, *L. tropica*, and *L. infantum*. By analyzing and comparing minicircle sequences from publicly available databases as well as our in-house data set, we identified conserved motifs upstream of the canonical conserved domain of minicircles, allowing us to design novel primers targeting these three species. All primers used for TIM tests, including degenerate positions, are listed in Table 1, and their locations within the conserved minicircle domain, compared to those of the Noyes test, are highlighted in Fig. 1C. To minimize non-specific amplifications, we carefully limited the number of degenerate nucleotides. The CSB2XF and CSB1XR primers from the Noyes assay contain four and three degenerate positions, respectively, resulting in 24 possible primer variants for the first PCR step. In contrast, the optional TIM1 pre-amplification step employs three primers (T1F1, T1F2, and T1R) with only two degenerate positions, reducing the number of potential primer variants to just four. Given that *L. tropica* minicircles display high sequence divergence from those of *L. infantum* and *L. major*, we had to design *L. tropica*-specific primers (T1F2 and T2R2).

The TIM assay principle is illustrated in Fig. 1B, and its comparison with other diagnostic methods used in Morocco is detailed in Table 1. Briefly, it consists of three distinct procedures (TIM1, TIM2, and TIM3), which can be selected based on the experimenter’s specific needs. The TIM1 PCR is an optional nested step dedicated to pre-amplify kDNA from the conserved domain of minicircles. PCR products from TIM1, diluted to 1:10, can then be used directly for TIM2 or TIM3 analyses as classical nested PCR procedures. TIM2 enables rapid screening of clinical samples within a single PCR. Since TIM2 is a multiplex PCR combining the three primers T2R1, T2R2, and T2F, it will generate a unique amplification product of approximately 800 bp for *L. tropica*, 770 bp for *L. infantum*, and 660 bp for *L. major*. In some cases, however, the discrimination between *L. infantum* and *L. tropica* species-specific bands might be challenging on agarose gels, as it is with the Noyes test. For that, we provide the option to split the TIM2 primers into two separate tests, collectively named TIM3: one for distinguishing *L. major* from *L. infantum* (TIM3 Lm/Li) using the T2R1 and T2F primers, and another specific for *L. tropica* (TIM3 Lt) using the T2R2 and T2F primers (Fig. 1B). Altogether, these flexible options enable researchers to optimize their analysis, either by enhancing sensitivity (TIM1), speed (TIM2), or improving species discrimination (TIM3).

### Comparison of TIM with ITS1-RFLP and kDNA nested PCR

**Fig 2:**
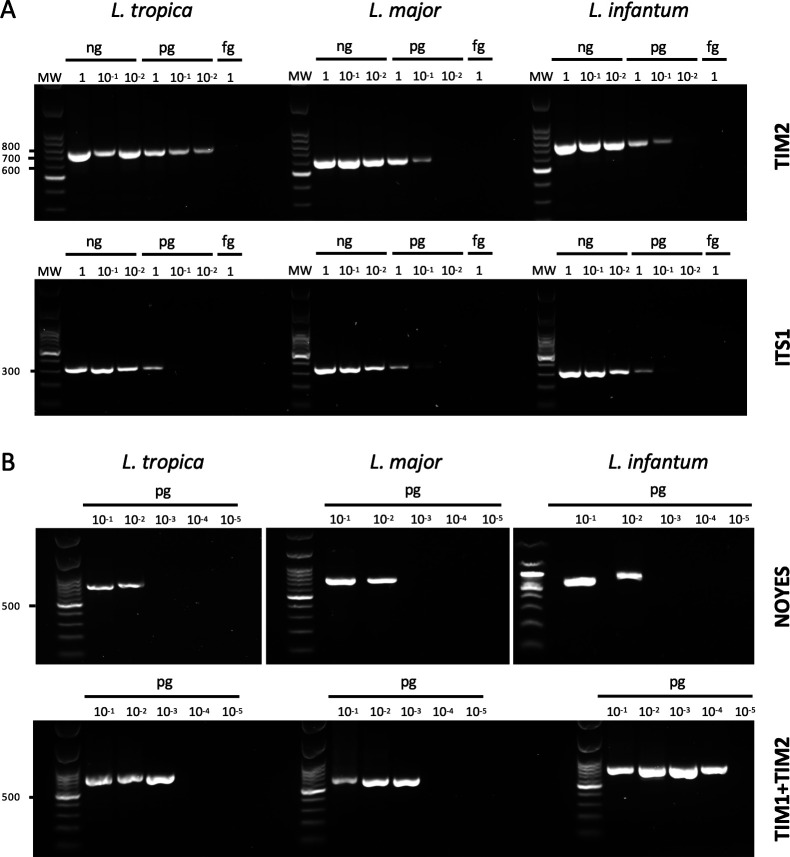
Sensitivity of TIM compared to ITS1-RFLP and Noyes diagnostic procedures on serial dilution of *L. tropica*, *L. major*, and *L. infantum* genomic DNA from Morocco strains isolated from patients. (**A**) Comparison of amplification products obtained with TIM2 and ITS1 PCRs from serial dilutions of DNA (from 1 ng to 1 fg). (**B**) Amplification products obtained with nested PCR from Noyes and TIM1 + TIM2 from serial dilution of DNA starting from 0.1 pg to 0.01 fg.

To evaluate the performance of TIM tests compared to conventional methods (ITS1*-*RFLP and kDNA nested PCR), we performed serial dilutions from 1 ng of DNA extracted from representative *L. tropica*, *L. major*, and *L. infantum* strains isolated from patients. As illustrated in Fig. 2A, the multiplex TIM2 assay demonstrates overall higher sensitivity and performance than ITS1 for detecting *L. major*, *L. infantum*, and *L. tropica* strains DNA. Given that the optional nested step of TIM1 is designed to further enhance sensitivity, we compared it to the widely used nested technique described by Noyes et al. (18). To this end, serial DNA dilutions were performed starting from 0.1 pg. As shown in Fig. 2B, the combined TIM1 + TIM2 assays outperform the Noyes nested method in sensitivity across all tested *Leishmania* species, with the greatest improvement observed for *L. infantum*. Overall, these results confirm that the new TIM design successfully achieves its primary objective, i.e., enhancing detection sensitivity for *L. infantum*, *L. major*, and *L. tropica* species compared to conventional diagnostic methods currently used in Morocco.

### Using the TIM3 assay for species discrimination and detection of mixed DNA

**Fig 3:**
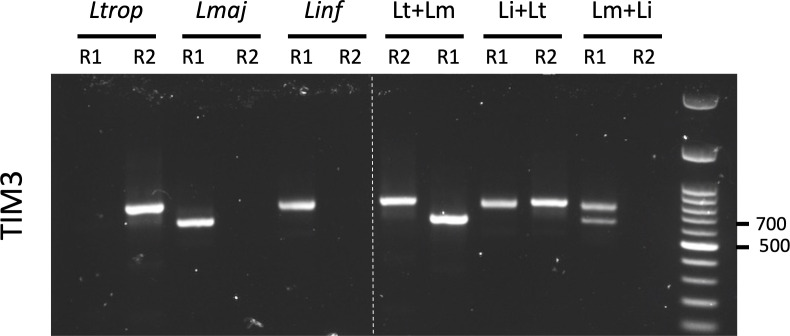
Application of TIM3 for the differentiation of *Leishmania* species and detection of mixed DNA samples. TIM3 was performed using either the primer pair T2F + T2R1 for the detection of *L. infantum* and *L. major* (R1), or T2F + T2R2 for the detection of *L. tropica* (R2). PCR reactions using either R1 or R2 primer sets were conducted on 0.1 ng of purified DNA from individual *Leishmania* species (*L. tropica*, *L. major*, and *L. infantum*) or from equimolar mixtures of two species (Lm + Li, Li + Lt, and Lm + Lt). Lm: *L. major*, Lt: *L. tropica*, and Li: *L. infantum*.

As previously mentioned, distinguishing between the diagnostics bands of *L. tropica* (800 bp) and *L. infantum* (770 bp) on agarose gels remains challenging when using either the Noyes or TIM2 procedures. Furthermore, the ability to detect co-infections or interspecific *Leishmania* hybrids by PCR would be a significant advantage for any diagnostic test, with implications for both clinical management and epidemiological surveillance. To determine whether the TIM3 assay can address these limitations, we evaluated its ability to detect artificial DNA mixtures of *L. major*, *L. tropica*, and *L. infantum* species, as well as its accuracy in discriminating *L. infantum* from *L. tropica* species. To this end, pure and equimolar DNA mixtures for the three *Leishmania* species were prepared and analyzed with both TIM3-R1 (specific to *L. major* and *L. infantum*) and TIM3-R2 (specific to *L. tropica*) assays, using an equal DNA input of 0.1 ng per condition. As shown in Fig. 3, TIM3 enabled clear differentiation of all tested conditions. Pure DNA from each species produced distinct bands at the expected sizes, while mixed samples were distinctly and unambiguously resolved. Notably, only TIM3 was able to identify *L. tropica + L. infantum* mixtures since *L. infantum* restriction fragments overlap those of *L. tropica* in the ITS1-RFLP method, while the amplicons from both species are too similar in size to be resolved by agarose gel in the Noyes assay (see Fig. 1A). Altogether, these results illustrate that TIM3 enables straightforward and specific differentiation of the three *Leishmania* species circulating in Morocco in a single assay and can further accurately assign each species in equimolar DNA mixtures.

### Evaluation of TIM on clinical samples

**Fig 4:**
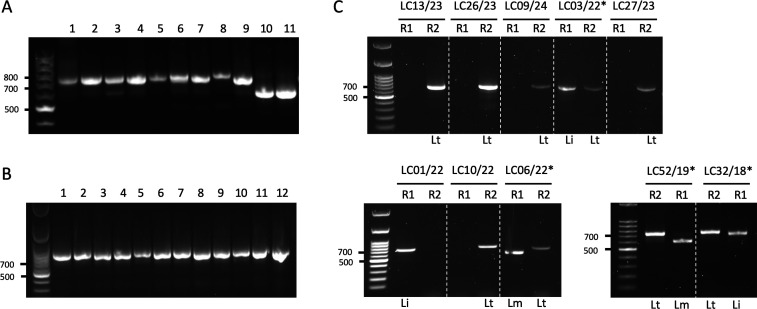
Evaluation of TIM2 and TIM3 assays on clinical samples. (**A**) Evaluation of TIM2 on 11 cutaneous clinical samples previously confirmed positive by the ITS1-RFLP method. (**B**) Evaluation of TIM2 on 12 positive cutaneous clinical samples collected from a *L. tropica* endemic focus. (**C**) Representative TIM3 assays performed on clinical samples from the Ibn Rochd hospital in Casablanca. TIM3 was carried out using either T2F + T2R1 primers for the detection of *L. infantum* and *L. major* species (R1), or with T2F + T2R2 for *L. tropica* parasites (R2). *: hybrid profiles.

To evaluate the efficiency of TIM in detecting and genotyping parasite DNA from CL clinical samples, we first analyzed 11 clinical samples previously identified as positive for different *Leishmania* species by ITS1-RFLP assays (Fig. 4A), as well as 12 *Leishmania* clinical samples positive by ITS1-RFLP collected from a *L. tropica* endemic focus (Fig. 4B). TIM2 assay successfully detected *Leishmania* DNA in 100% of the analyzed samples (*n* = 23) using a single PCR test. We then examined three CL suspicion cases that had previously tested negative for *Leishmania* by ITS1 PCR (Fig. S1). TIM3 analysis of these samples yielded a positive signal in two out of three cases, both identified as *L. infantum*. To evaluate whether TIM1 preamplification could validate or refine these results, we applied the combined TIM1 + TIM3 assay to the same samples. Interestingly, if samples 1 and 2 were confirmed as *L. infantum*, the third sample became positive for *L. tropica* (Fig. S1). These results illustrate that TIM3 outperforms the ITS1-RFLP method, and TIM1 significantly improves assay sensitivity by allowing the recovery of false-negative diagnostics. In a second step, we performed a comparative analysis between the ITS1-RFLP method and TIM on clinical samples obtained at the Dermatology Department of the Ibn Rochd University Hospital Center in Casablanca. The supplementary table lists the epidemiological and clinical information of the 44 CL patients included in the study. The patient’s sample comprised 25 females (56.8%) and 19 males (43.2%), with ages ranging from 7 months to 85 years (median: 43 years). Incubation periods (estimated period between the patient’s stay in a known endemic area and lesion appearance) varied from 3 weeks to 10 months. Lesions were predominantly single (*n* = 32), with 12 patients presenting multiple lesions (2–6). Regarding molecular typing, seven samples that were negative by ITS1-RFLP were found positive by TIM2 or TIM3 assays. In addition, TIM unequivocally identified the *Leishmania* causative species for three patients for whom the ITS1-RFLP PCR technique had been inconclusive and only allowed the diagnosis of *Leishmania* genus (Table S1). Furthermore, three samples that had been ambiguously classified as *L. tropica* and *L. infantum* by ITS1-RFLP, due to poor resolution on agarose gels, were correctly reassigned by TIM as *L. infantum* and *L. tropica*, respectively. Finally, four samples were identified as negative by all tested methods, including the nested TIM1 + TIM3 assay (Table S1). Figure 4C illustrates the results of representative TIM3 tests, demonstrating the ability of this technique to provide an accurate and conclusive species diagnosis, notably between *L. tropica* and *L. infantum* species. Interestingly, TIM3 further revealed five mixed profiles characterized by the coexistence of two species-specific mitochondrial genotypes, each combining *L. tropica* with either *L. major* or *L. infantum* (Fig. 4C).

## DISCUSSION

The search for an ideal *Leishmania* diagnostic method has been a long-standing challenge.

This complexity results notably from the large number of parasite species capable of infecting humans (1). To date, no diagnostic method combines high sensitivity and specificity while being universally applicable to all pathogenic *Leishmania* species (19). In this study, we chose to develop a tailored diagnostic test suited to the context of CL in North Africa, particularly in Morocco. We also prioritized PCR as it is a well-established and highly sensitive technique, widely adopted in laboratories and hospitals worldwide, allowing for easy and rapid implementation (13). Given their extremely high copy number per parasite, kDNA minicircles are well recognized as ideal molecular targets for diagnosis (up to 10,000 copies per parasite vs 40–200 copies for rDNA) (20, 21). Currently, the two most used approaches to diagnose and genotype *Leishmania* species using conventional PCR techniques are the ITS1-RFLP method on rDNA and the nested-PCR on kDNA. While widely adopted, these methodologies suffer from certain limitations that may lead to inconclusive genotyping results. Species identification with the Noyes test is only based on size discrimination, which can be an issue in areas like Morocco, where endemic *Leishmania* species, i.e., *L. tropica* and *L. infantum*, possess nearly identical amplification product sizes. Similarly, with the less sensitive ITS1-RFLP approach that needs to combine PCR and a restriction digestion step, differentiation between some species relies solely on a faint additional band at very low molecular weight (100 bp), which can be overlooked in poorly amplified restriction profiles.

**Fig 5:**
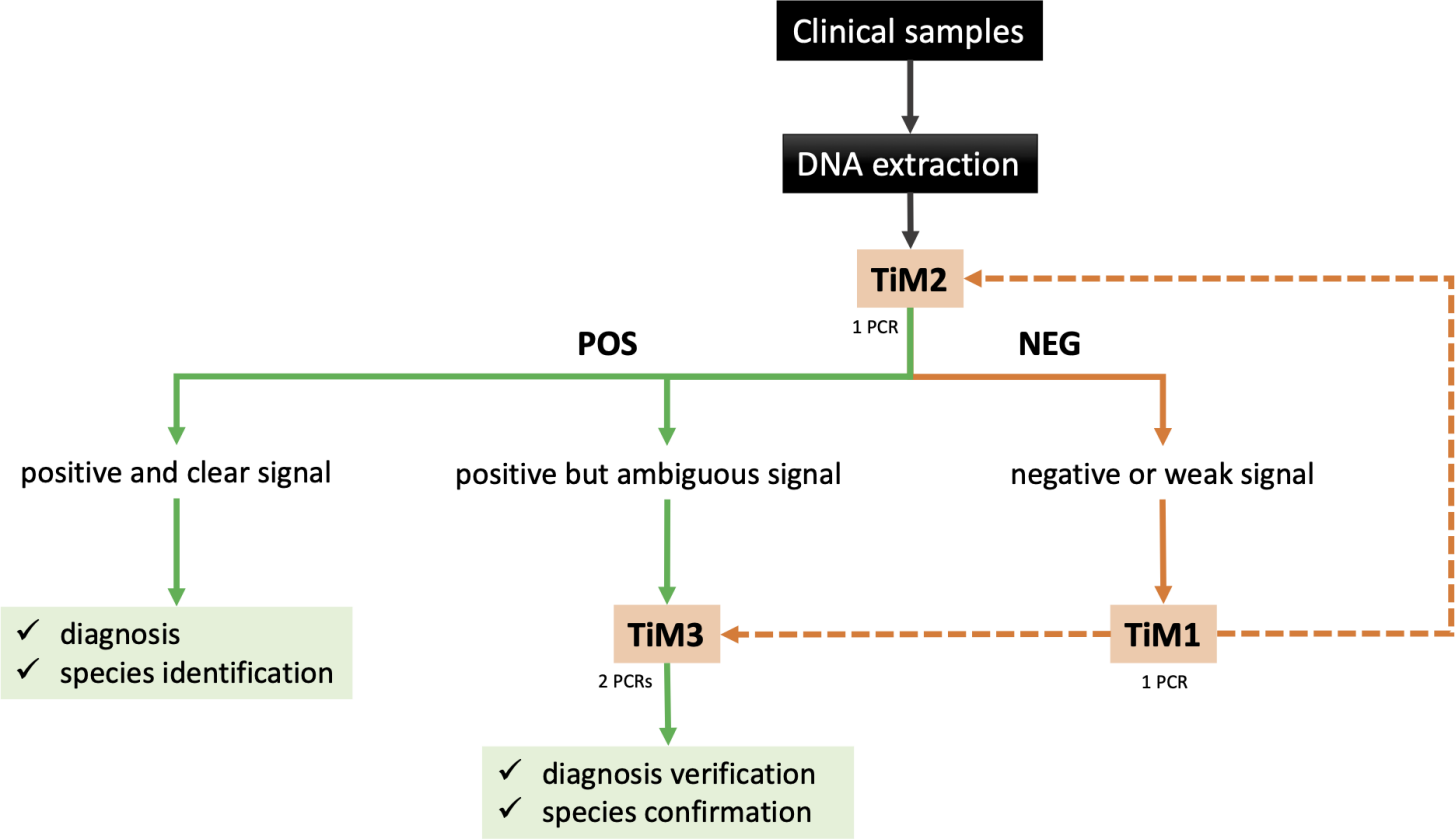
Proposed workflow for the routine diagnostic application of TIM assays. The scheme outlines the recommended steps for sample processing, PCR assay selection (TIM1, TIM2, or TIM3), and interpretation of results.

The newly developed TIM assays successfully detected *Leishmania* DNA in all positive samples, achieving 100% detection regardless of sample type (dermal aspirations, swabs, or biopsies). The enhanced sensitivity of the TIM PCR assays enabled making up infections that would likely be missed by conventional methods (Fig. S1). As demonstrated in the supplementary figure, TIM1 pre-amplification further increases assay sensitivity and helps distinguish true negative samples from those with low parasite loads. TIM tests offer, therefore, high versatility with applications that can be adapted to various diagnostic needs. A suggested workflow for the practical application of TIM in the context of CL is presented in Fig. 5. Since TIM assays offer better sensitivity toward *L. infantum* than conventional methods tested here, we can assume that they are also well adapted for the diagnosis of mammalian infection in the context of VL. Nevertheless, pre-analytic steps, i.e., sampling and DNA extraction procedures, remain crucial steps to perform accurate diagnosis (22). Of note, we observed that diluting clinical sample DNA (e.g., at a 1:10 ratio) may enhance diagnostic performance and species identification with TIM2 or TIM3, particularly when DNA is extracted using the phenol/chloroform method.

A distinctive feature of the TIM3 test is its capacity to identify mixed DNA profiles in CL clinical samples (observed in approximately 10% of cases). These profiles may correspond either to co-infections involving different *Leishmania* species or to natural interspecific hybrids. Interestingly, all these mixed profiles (*n* = 5) involved *L. tropica*, yet no clear associations were observed with patient age or clinical presentation. From an epidemiological perspective, the reported geographical expansion of *L. tropica* into areas historically endemic for *L. major* and *L. infantum* is consistent with these observations. Moreover, all the regions where patients with mixed profiles were reportedly infected are characterized by the co-circulation of multiple *Leishmania* species (4, 5, 23, 24). Several cases of mixed *Leishmania* species infections have been reported in the literature (25) and are very likely to be underdiagnosed. Natural co-infections involving multiple species or genotypes may give rise to cooperative or competitive interactions between strains, potentially resulting in complex dynamics with unforeseen consequences in terms of virulence and pathogenicity. One recognized outcome of such mixed infections in sandfly vectors is, however, the generation of hybrid parasites. The existence of hybridization among *Leishmania* species or strains has been known for decades, with observations made under both natural and experimental conditions (26–28). Hybridization in *Leishmania* occurs when two genetically distinct strains or species recombine, typically within the sandfly vector. This process occurring at both inter- and intraspecific levels leads to the formation of parasites with new genetic and phenotypic traits, which may potentially impact parasite virulence, pathogenicity, transmissibility by vectors, or drug susceptibility (29, 30). Recent genome-scale analyses provided evidence of recombination between New World *Leishmania* species in Peru, resulting in full genome hybrids (31). Interestingly, the mitochondrial genome of these hybrid strains consisted of homogeneous uniparental maxicircles, whereas minicircles originated from both parental species. These results, therefore, support the minicircle-based approach as a suitable strategy for detecting interspecific hybrids. Distinguishing hybridization from co-infection in clinical samples is challenging and usually demands multilocus or whole-genome sequencing of species-specific nuclear markers. While these methods provide high-resolution insights, they are time-consuming and costly, making them inaccessible in most routine diagnostic settings. Nevertheless, the ability of TIM assays to detect mixed profiles directly from clinical samples using a simple PCR test can provide valuable insights into *Leishmania* transmission dynamics and help monitor the potential occurrence of co-infections or hybridization under natural conditions.

By combining high sensitivity, flexibility, and species-level resolution, the TIM diagnostic toolbox aligns closely with the WHO TPP for dermal leishmaniasis to support both routine diagnosis and epidemiological surveillance. Finally, it is worth emphasizing that the TIM approach is not limited to the Moroccan context. It holds significant potential for application in other countries across the Mediterranean basin, particularly in North Africa, where *L. tropica*, *L. major*, and *L. infantum* species are likewise endemic and may overlap geographically.

## SUPPLEMENTAL MATERIAL

Supplemental material spectrum.03447-25-s0001.pdf Fig. S1; Table S1.

ASM does not own the copyrights to Supplemental Material that may be linked to, or accessed through, an article. The authors have granted ASM a non-exclusive, world-wide license to publish the Supplemental Material files. Please contact the corresponding author directly for reuse.
