# Supplementary material for: TIM, a novel molecular toolbox for the detection and identification of Leishmania species in Morocco
Source: Microbiol Spectr. 2025 Dec 4;14(1):e03447-25. doi: 10.1128/spectrum.03447-25 (PMC12772242; doi:10.1128/spectrum.03447-25)
Supplement: Supplemental material — Fig. S1; Table S1. [file spectrum.03447-25-s0001.pdf]

Supplementary material for

« TIM, A Novel Molecular Toolbox for the Detection and Identification of *Leishmania* Species in Morocco ».

El Idrissi Saik Imane, Lemkhayar Kaltoum, Mhaidi Idris, El Mazini Sara, Chiheb Soumiya, Bañuls Anne-Laure, Lemrani Meryem, Riyad Myriam, Vergnes Baptiste

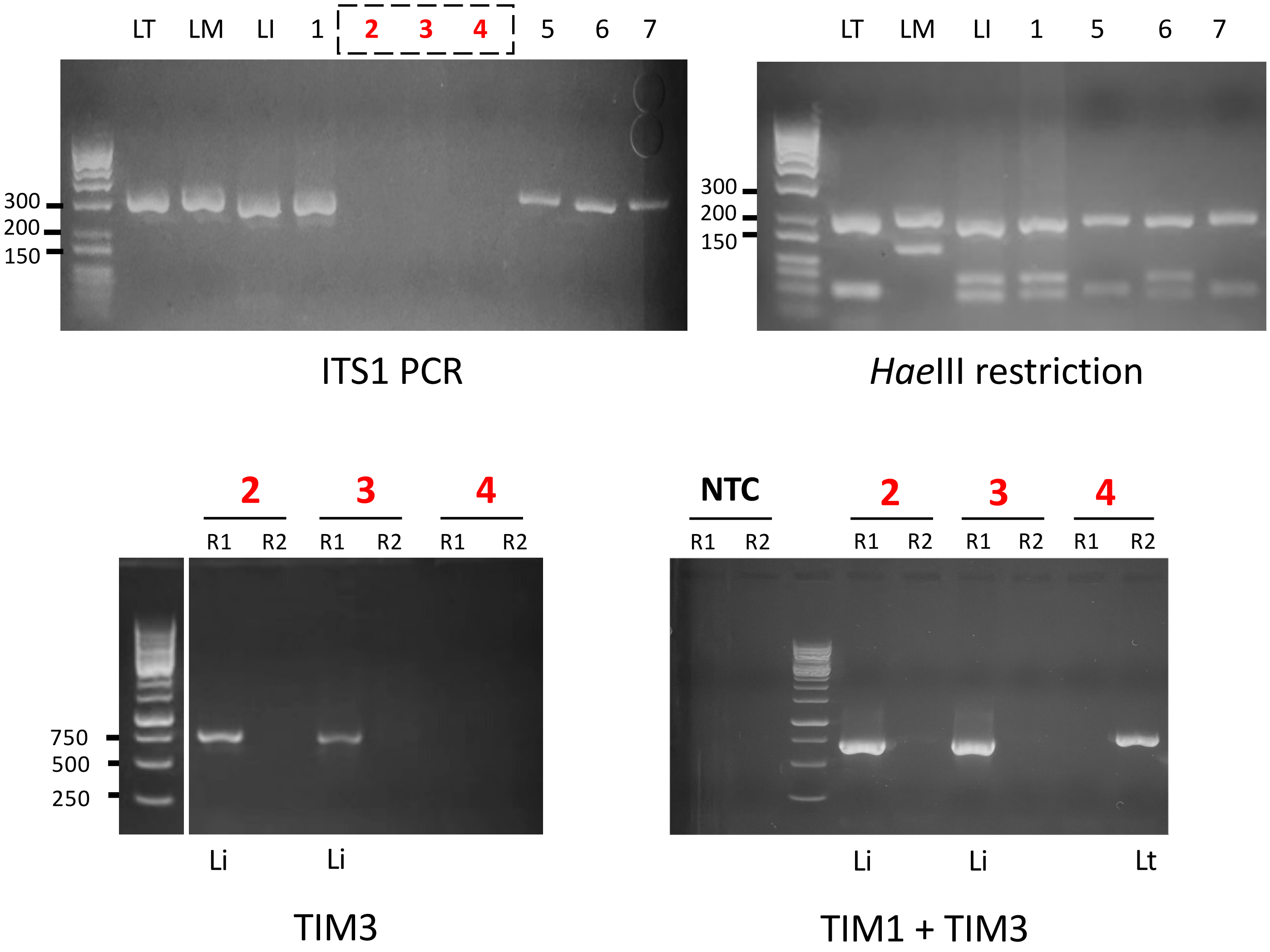

Supplementary figure. Enhanced sensitivity of parasite detection using the nested TIM1 approach. Seven CL clinical samples were initially analyzed using the ITS1-RFLP method, alongside reference strains: *L. tropica* (LT), *L. major* (LM), and *L. infantum* (LI). Positive PCR signals were observed for samples 1 and 5–7. *Hae*III restriction profiles of the amplified products are shown on the right, enabling accurate species identification. Samples 2 to 4, which tested negative by ITS1-PCR, were subsequently analyzed using the TIM3 approach. Samples 2 and 3 produced PCR signals corresponding to the *L. infantum*-specific amplicon (lanes R1). These three samples were reanalyzed using the combined TIM1+TIM3 protocol, which confirmed *L. infantum* detection in samples 2 and 3, and successfully identified *L. tropica* in sample 4. NTC: no template control.

Code	Age	Gender (F, M)	Travel to or residence in CL endemic regions	Incubation Duration	No. of lesions	ITS1-RFLP	TIM (2 or 3)
LC11/18	54 y	F	Draa-Tafilalet	1 m	1	LT	LI
LC12/18	1.5 y	F	Souss-Massa	1.5 y	2	LT	LT
LC16/18	19 y	M	Draa-Tafilalet	N/D	1	neg	LT
LC21/18	59 y	F	Draa-Tafilalet	2 m	1	LT	LT
LC32/18	33 y	M	Beni Mellal-Khenifra	6 m	1	LT	LT+LI
LC38/18	85 y	F	Draa-Tafilalet	1 m	1	neg	LT+LM
LC41/18	51 y	F	Marrakech-Safi	N/D	1	L	LM
LC44/18	6 y	F	Draa-Tafilalet	1 m	1	LM	LM
LC45/18	29 y	M	Draa-Tafilalet	2 m	1	neg	LM
LC47/18	23 y	F	Draa-Tafilalet	1 m	1	neg	LM
LC49/18	48 y	F	Draa-Tafilalet	1 m	1	neg	LM
LC02/19	27 y	F	Draa-Tafilalet	3 m	1	LM	LM
LC52/19	1 y 3 m	M	Draa-Tafilalet	N/D	1	LT	LT+LM
LC14/20	44 y	M	Draa-Tafilalet	1 m	1	neg	LM
LC01/22	43 y	M	Fes-Meknes	N/D	1	LI	LI
LC03/22	25 y	M	Beni Mellal-Khenifra	1 m	1	neg	LT+LI
LC04/22	16 y	M	Souss-Massa	7 m	1	LT	LT
LC06/22	55 y	F	Marrakech-Safi	6 m	3	LT	LT+LM
LC07/22	70 y	M	Marrakech-Safi	6 m	1	LT	LT
LC10/22	30 y	F	Fes-Meknes	N/D	1	LI	LT
LC13/23	55 y	F	Marrakech-Safi	6 m	3	LT	LT
LC17/23	10 y	M	Casablanca-Settat	6 m	1	LT	LT
LC18/23	61 y	F	Tanger-Tetouan-Al Hoceima	4 m	1	LI	LI
LC20/23	60 y	M	Beni Mellal-Khenifra	1 m	1	LT	LT
LC21/23	54 y	F	Marrakech-Safi	N/D	2	LI	LI
LC22/23	5 y	F	Fes-Meknes	1 m	1	LI	LI
LC23/23	12 y	M	Draa-Tafilalet	10 m	1	LT	LT
LC24/23	7 y	M	Casablanca-Settat	N/D	1	LT	LT
LC26/23	69 y	F	Tanger-Tetouan-Al Hoceima	3 m	2	LT	LT
LC27/23	63 y	F	Marrakech-Safi	2 m	1	LT	LT
LC28/23	51 y	F	Casablanca-Settat	N/D	1	LT	LT
LC01/24	12 y	M	Souss-Massa	6 m	1	LT	LT
LC04/24	68 y	F	Draa-Tafilalet	2 m	4	LM	LM
LC05/24	61 y	F	Marrakech-Safi	N/D	3	LT	LT
LC06/24	62 y	M	Marrakech-Safi	2 m	4	LT	LT
LC07/24	63 y	M	ND	N/D	1	L	LT
LC08/24	13 y	M	Draa-Tafilalet	2 m	2	LM	LM
LC09/24	7 m	F	Draa-Tafilalet	1 m	6	LT	LT
LC10/24	72 y	F	Fes-Meknes	N/D	1	LT	LI
LC13/24	49 y	M	Beni Mellal-Khenifra	2 m	4	L	LT
LC23/24	35 y	M	Dakhla-Oued Ed-Dahab	N/D	3	neg	neg (TIM1+TIM3)
LC24/24	57 y	F	Beni Mellal-Khenifra	3 w	1	neg	neg (TIM1+TIM3)
LC25/24	13 y	F	Marrakech-Safi	1 m	1	neg	neg (TIM1+TIM3)
LC26/24	62 y	F	Drâa-Tafilalet	9 m	1	neg	neg (TIM1+TIM3)

Supplementary table. Epidemiological and clinical information of CL suspected patients and molecular typing results with ITS1-RFLP and TIM assays.

L: *Leishmania spp.*, LM: *L. major*, LT: *L. tropica*, LI: *L. infantum*.

y: Years, m: Months ; F: Female, M: Male ; N/D: not defined ; N/R: not relevant ; neg: negative PCR
